# Assessing the validity of indicators of the quality of maternal and newborn health care in Kenya

**DOI:** 10.7189/jogh.06.010405

**Published:** 2016-06

**Authors:** Ann K Blanc, Charlotte Warren, Katharine J McCarthy, James Kimani, Charity Ndwiga, Saumya RamaRao

**Affiliations:** 1 Population Council, New York, NY, USA; 2 Population Council, Washington D.C., USA; 3 Population Council, Nairobi, Kenya

## Abstract

**Background:**

The measurement of progress in maternal and newborn health often relies on data provided by women in surveys on the quality of care they received. The majority of these indicators, however, including the widely tracked “skilled attendance at birth” indicator, have not been validated. We assess the validity of a large set of maternal and newborn health indicators that are included or have the potential to be included in population–based surveys.

**Methods:**

We compare women’s reports of care received during labor and delivery in two Kenyan hospitals prior to discharge against a reference standard of direct observations by a trained third party (n = 662). We assessed individual–level reporting accuracy by quantifying the area under the receiver operating curve (AUC) and estimated population–level accuracy using the inflation factor (IF) for each indicator with sufficient numbers for analysis.

**Findings:**

Four of 41 indicators performed well on both validation criteria (AUC>0.70 and 0.75<IF<1.25). These were: main provider during delivery was a nurse/midwife, a support companion was present at birth, cesarean operation, and low birthweight infant (<2500 g). Twenty–one indicators met acceptable levels for one criterion only (11 for AUC; 9 for IF). The skilled birth attendance indicator met the IF criterion only.

**Interpretation:**

Few indicators met both validation criteria, partly because many routine care interventions almost always occurred, and there was insufficient variation for robust analysis. Validity is influenced by whether the woman had a cesarean section, and by question wording. Low validity is associated with indicators related to the timing or sequence of events. The validity of maternal and newborn quality of care indicators should be assessed in a range of settings to refine these findings.

Nearly 275 000 maternal deaths occurred globally in 2011, nearly all of which took place in low– and middle–income countries (LMIC) [1]. Most of these countries did not reduce maternal mortality to levels targeted in the Millennium Development Goals (MDG5) [1]. Progress has been hindered, in part, by a lack of reliable maternal health data, especially on maternal deaths [2]. Measurement challenges are particularly significant in LMIC with irregular and incomplete health system reporting.

To measure progress in maternal health, monitoring agencies have relied on tracking indicators proposed as measures of quality of care, such as the proportion of births attended by a skilled birth attendant, that are assumed to be strongly correlated with maternal mortality [3]. Such indicators are routinely assessed in population––based household survey programs, such as the Demographic and Health Surveys (DHS) and Multiple Indicator Cluster Surveys (MICS), in which female respondents report on events surrounding recent births [4]. Despite their widespread use, the majority of proposed quality of care indicators, including skilled birth attendance, have not been validated [1,5,6]. In fact, numerous researchers have noted the lack of correlation between these indicators and maternal mortality levels [5,7–9]. These researchers argue that information on the category of provider at birth is deficient as a measure of quality of care as it relies on assumptions about provider training and competence as well as access to essential supplies and equipment. It is important therefore to identify alternate indicators that describe the actual content of care, can be reported with accuracy, and have the potential to be included in routine data collection programs.

A growing, but still limited, body of research has examined the validity of indicators of the quality of care in the intrapartum and early postpartum period. To our knowledge, however, no study has yet reported on how accurately women can recall the skill level of their provider at birth, although there have been some attempts to look at data quality issues [10]. Furthermore, the few validation studies that have taken place have generally compared maternal self–reports with hospital records, which may be incomplete or inaccurate, or have been conducted in high–income settings, where maternal mortality rates are generally low [11–15].

To address this gap, this study assessed women’s ability to report on a set of quality of maternal and newborn health care indicators that are either currently in use or have the potential to be included in routine survey–based data collection. In spite of its limitations, it seems likely that the “skilled birth attendance” indicator will continue to be used and so we assess how accurately women report on the skill level of their provider during delivery. We compare women’s self–reports of maternal and newborn care received against third party observations during labor and delivery. Finally, we provide suggestions for modifications to data collection procedures that could improve the measurement of maternal and newborn health care.

## METHODS

### Study sites

Validation exercises were conducted in two high volume public hospitals located in Kisumu District and Kiambu District in western and central Kenya, respectively. According to the 2014 Kenya DHS, nationally, 61% of births in the five years preceding the survey were delivered in a health facility; in Kisumu and Kiambu districts the prevalence was 70% and 93%, respectively [16]. Facility–based delivery is less likely among older women, those who have lower education, are poorer, or reside in rural areas [16]. Fertility levels among women in the two districts are lower than the national rate, with the total fertility rate in Kisumu at 3.6 births per woman and in Kiambu at 2.7, compared with 3.9 nationally [16].

### Data collection

Data collection took place from July to September 2013. All pregnant women aged 15 to 44 who were admitted to a study facility maternity unit and in early labor were invited to participate. Participants included eligible women who underwent labor and delivery and were able to provide consent.

Our reference standard for validity analysis is data collected by trained researchers who observed providers in the maternity admission room and labor and delivery rooms using a structured checklist–type form. Observers were registered Kenyan nurse/midwives with at least three years of experience in a maternal and newborn health unit and previous research experience. Observations were used as the reference standard as they reflected all facets of caregiving including events related to the birth itself as well as interactions between the women and provider, before, during and up to one hour after delivery. In the few cases in which clarification was needed (eg, in the event the mother and infant were taken into separate rooms, the observer remained with the mother) observations were supplemented by checking facility records and by asking providers.

Exit interviews with women took place prior to hospital discharge. Data collectors who were degree holders in a social science interviewed women using a structured questionnaire. Interview questionnaires were translated into Kiswahili, Dholuo and Kikuyu and were administered in the woman’s language of preference.

All data collectors received four days of intensive training on the study procedures, the rationale behind each element of the client questionnaire and observation checklist to ensure full understanding of the instrument components, and how to record responses and observations.

### Ethical review

Written informed consent was obtained from all participants and their attending providers prior to participation. All women and providers were provided with a description of the study and procedures, including their right to refuse participation at any time. In Kenya, pregnant adolescents between ages 15–17 years are considered “emancipated minors” and their written informed consent was also obtained [17–19]. Staff who provide labor and delivery care were identified by the hospitals’ obstetrics and gynaecology director, and approached for recruitment and consent. No providers refused participation.

Prior to participant enrollment, the study protocol was approved by the ethical review committees of the Population Council and the Kenya Medical Research Institute (KEMRI).

### Indicator selection

To identify indicators to be validated, a landscaping scan of published and grey literature was conducted from April to July 2012. The scan focused on indicators of the quality and content of care received during labor and delivery and the health outcomes related to this period [20]. Indicators were included if they were currently in use or proposed for use in household survey programs such as the DHS and MICS or reflected standard practices of maternal and newborn labor and delivery care. The scan yielded a list of 285 indicators. This list was assessed by a group of public health experts specializing in maternal health to select a set of 80 indicators for validity testing. Indicators were selected on the basis of their wide use and/or potential to assess the critical elements of maternal and newborn care during the initial assessment of the woman, the first, second and third stages of labor, and immediate postnatal period.

### Analysis

Sample size was calculated assuming 50% prevalence for all indicators, given that some harmful practices would rarely occur, and some beneficial practices would almost always occur, at 60% sensitivity ±6% precision, 70% specificity ±6% precision, with type 1 error set at α = 0.05 assuming a normal approximation to a binomial distribution. These specifications imply a minimal sample size of 500, which was increased to 600 women to allow for 20% attrition in a separate study to re–interview women approximately one year following delivery.

Statistical analysis was performed using Stata Version 12 [21] to assess indicator accuracy at the individual and population level. For individual–level reporting accuracy, we calculated the sensitivity (ie, true positive rate) and specificity (ie, true negative rate) of indicators by constructing two–by–two tables for each indicator that had at least five counts per cell [22].

Missing pairwise data were excluded. To summarize the accuracy of each indicator, we quantified the area under the receiver operating curve (AUC), which plots the sensitivity (ie, true positive rate) of each indicator against its false positive rate (1–specificity). To measure uncertainty associated with validity, we estimated 95% confidence intervals (CI), assuming a binomial distribution. In practice, the AUC represents the “average accuracy of a diagnostic test” [23,24]. AUC values range from 1.0 (perfect classification accuracy) to 0 (zero accuracy). An AUC value of 0.5 is the equivalent of a random guess.

To assess the population–based validity of indicators, we estimated the inflation factor (IF), also known as the Test to Actual Positives (TAP) ratio [25]. The IF reflects the prevalence of the indicator as it would be reported by women in a survey after accounting for sensitivity and specificity (Pr) divided by the true prevalence (ie, observer report) (P). By comparing the ratio of the estimated survey–based prevalence to its true prevalence, we calculated the degree to which each indicator would be over or under–estimated by women’s self–report (IF = Pr/P) [25,26].

The prevalence of women’s self–report in a survey (Pr) is calculated by applying each indicator’s estimated sensitivity (SE) and specificity (SP) to its true prevalence (P), using the following equation: Pr = P × (SE+SP−1)+(1−SP) [26]. We caution that the estimated survey–based prevalence is dependent on the observed prevalence of the indicator. Therefore, IF estimates reflect the magnitude of over or under–estimation in the study setting. To illustrate the implications of the IF estimates for other contexts in which the true prevalence is different from our study setting (eg, outside of a hospital facility), we model the estimated survey prevalence for select indicators across all possible coverage levels (ie, true prevalence ranging from 0 to 100%) using the above equation [27].

We categorized individual–level reporting accuracy as high (AUC>0.70), moderate (0.60<AUC<0.70), and low (AUC<0.60) [22] and the degree of bias reflected by the IF as low (0.75<IF<1.25), moderate (0.50<IF<1.5) and large (IF<0.50 or IF>1.5) [11]. In order to summarize indicator validity in terms of both individual and population–level accuracy, we considered indicators with high AUC and low IF to have high overall performance [22].

### Role of the funding source

The funders of the study had no role in data collection, analysis, interpretation or writing of the study results, or decision to submit for publication.

## RESULTS

### Sample description

**Figure 1:**
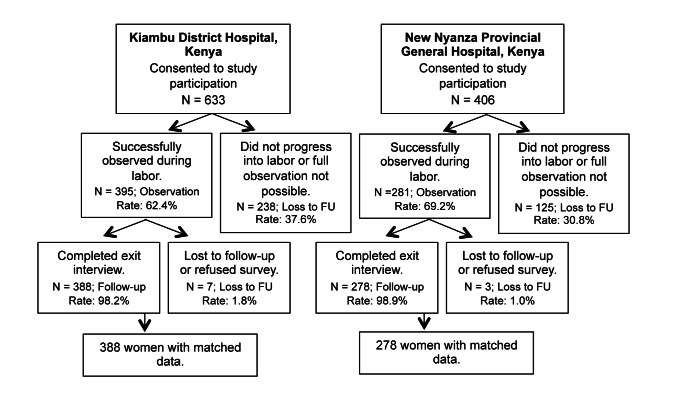
Participant response rates.

1039 women admitted to the maternity unit at participating study facilities were recruited to participate. Of those, 676 women were observed (Kiambu = 395, Kisumu = 281). Approximately one–third of women were not observed because they were not in labor but required monitoring on the antenatal ward, did not progress into labor, or they progressed rapidly into labor and full observation was not possible (**Figure 1**). Fourteen women who were observed did not participate in the exit interview.

Participants’ background characteristics and differences by facility location are presented in **Table 1**. The majority of women were under age 25 with fewer than two prior births, married, and with no or primary education. A greater percentage of Kisumu participants were never married, while fewer were married/living together or separated/widowed.

**Table 1:** Sample background characteristics by facility location

		% total sample (N = 662)		% Kiambu (N = 388)	% Kisumu (N = 274)	*P*–value*
**Age in years:**						0.504
15–19		14.7		12.4	17.9	
20–24		40.8		41.8	39.4	
25–29		29.9		30.2	29.6	
30–34		8.6		9.3	7.7	
35–39		5.6		5.9	5.1	
40–45		0.5		0.5	0.4	
**Prior parity (total number of live births):**						0.435
0			50.2	49.7	51.3	
1			26.5	28.8	22.9	
2			14.0	13.2	14.9	
3			6.0	5.2	7.3	
4 or more			3.3	3.1	3.6	
**Educational attainment:**						0.001
None	10.3			10.3	10.2	
Primary	44.0			45.9	41.2	
Secondary	29.5			33.2†	24.1†	
Higher	16.3			10.6†	24.5^†^	
**Marital status:**						0.001
Single, never married	14.7			9.8†	21.5†	
Married/living together	83.4			87.6†	77.4†	
Separated/widowed	2.0			2.6†	1.1†	
**Type of delivery:**						0.679
Vaginal	86.6			87.0	85.9	
Cesarean section	13.4			13.0	14.1	

*Based on χ^2^ test comparing facility locations, statistically significant at *P* < 0.05.

†Statistically significant pairwise comparisons using the Holm–Bonferroni correction to adjust for multiple comparisons.

### Validation results

The full list of indicators selected for validity testing is presented in **Table 2**. The table provides the prevalence for each indicator as reported by women and observers, which, for some indicators, varied substantially. For example, 73% of women reported that the provider(s) washed his or her hands or used antiseptic before any initial examination, while 27% of observers recorded that this took place. “Don’t know” responses were minimal for most indicators. However, four indicators for which the proportion of women who responded “Don’t know” exceeded 5% are reported in **Table 3**. Two of these indicators refer to the immediate postnatal period: whether the newborn was immediately dried after birth and whether the newborn was immediately wrapped in a towel. Having a cesarean section as opposed to a vaginal delivery was significantly associated with responding “Don’t Know” to both of these questions (OR = 15.3, 95% CI = 8.2–28.3, *P* < 0.001; OR = 2.7, 95% CI = 1.2–5.9, *P* = 0.016, respectively).

**Table 2:** List of indicators assessed and reported prevalence*

Indicator	N	Women’s self–report, prevalence (%)	Observer report, prevalence (%)	At least 5 counts/cell
**Initial client assessment:**				
Type of facility where gave birth (public hospital)	651	98.0	100.0	No
Referred to facility because of a problem	655	8.2	7.9	Yes
HIV status checked	659	25.2	94.7	No
Offered HIV test	660	8.3	1.7	No
Receives HIV test	654	8.6	9.3	No
Provider washes hands with soap and water or uses antiseptic before any initial examination	467	73.1	26.6	Yes
Takes blood pressure	654	93.4	87.0	Yes
Takes urine sample	654	5.7	1.4	No
Checks fetal heart rate with fetoscope/ultrasound	659	95.8	99.7	No
Wears high–level disinfected or sterile gloves for vaginal examination	658	99.9	99.9	No
**Provider respectful care:**				
Woman allowed to drink liquids/eat	624	66.8	42.0	Yes
Encourages/assists woman to ambulate during labor	644	87.0	77.5	Yes
Encourages/assists woman to assume different positions in labor	649	14.3	58.2	Yes
Woman allowed to have a support person present during labor and delivery	648	8.8	9.1	Yes
A support person is present at birth	644	3.7	4.8	Yes
**First stage of labor:**				
Induces labor by uterotonic (IV, IM, tablet)	630	10.8	4.6	Yes
Augments labor with uterotonic (by IV line, IM injection, or tablet)	625	39.2	22.4	Yes
Uterotonic received (to induce or augment labor)	619	43.8	27.1	Yes
Membranes ruptured (to induce or augment labor)	650	3.1	42.3	Yes
**Skilled birth attendance–main provider:**				
Main provider labor				
Skilled main provider labor†	649	89.9	92.6	Yes
Main provider labor–doctor or medical resident	649	9.6	0.5	No
Main provider labor–doctor (ob–gyn)	649	9.6	0.3	No
Main provider labor–medical resident	649	0.0	0.2	No
Main provider labor–medical intern	649	0.2	1.9	No
Main provider labor–nurse/midwife	649	80.2	92.1	Yes
Main provider labor–clinical officer	649	2.2	0.6	No
Main provider labor–facility support/ staff aide	649	0.2	0.3	No
Main provider labor–student nurse	649	2.3	2.8	No
Main provider labor–support companion	649	0.6	1.7	No
Main provider labor–no one or other	649	4.6	0.0	No
Main provider delivery				
Skilled main provider delivery†	644	94.3	92.9	Yes
Main provider delivery– doctor (ob–gyn) or medical resident	644	19.3	11.8	Yes
Main provider delivery– doctor (ob–gyn)	644	19.1	3.0	No
Main provider delivery–medical resident	644	0.2	8.9	No
Main provider delivery–medical intern	644	0.3	1.1	No
Main provider delivery–nurse/midwife	644	75.0	81.1	Yes
Main provider delivery–clinical officer	644	2.2	0.8	No
Main provider delivery–student nurse	644	2.2	4.8	Yes
Main provider delivery–no one or other	644	0.9	0.5	No
**Second and third stage of labor:**				
Episiotomy performed	545	22.9	18.2	Yes
Uterotonic administered within few minutes of delivery (via injection, IV medication, or oral/rectal tablets)	562	96.8	98.8	No
Uterotonic received 1–3 min after birth	552	96.9	81.5	No
Uterotonic received after delivery of placenta	552	59.1	2.4	Yes
Applies controlled cord traction	561	97.5	98.9	No
Performs uterine massage after delivery of placenta	558	88.4	98.6	No
Position of mother at birth–on back	645	94.7	99.8	No
Health provider wore gloves during delivery of baby	563	100.0	99.8	No
**Immediate postnatal newborn care:‡**				
Newborn given to mother immediately after birth	611	59.9	57.6	Yes
Newborn immediately dried with towel/cloth	594	96.1	99.5	No
Newborn placed immediately skin to skin on mother's chest§	602	78.9	16.3	Yes
Newborn immediately skin to skin on mother (2 item indicator)#	596	29.2	16.2	Yes
(Of newborns not on skin) Newborn wrapped with towel	86	90.7	91.9	No
Breastfeeding initiated within first hour of birth	551	76.4	53.0	Yes
Something other than breastmilk given to baby within first hour of delivery	572	1.9	1.1	No
Baby bathed within the first hour after birth	604	2.8	0.1	No
Baby weighed	635	99.8	100.0	No
Low birth–weight baby (<2500g)	579	6.7	7.8	Yes
High birth–weight baby (≥4500g)	579	1.0	1.0	No
3 elements of newborn care (immed. · dried + on skin + breastfed in first hour)	506	71.5	9.3	Yes
3 elements of newborn care (immed. · dried, 2 item skin–to–skin#, breastfed in first hour)	501	25.1	9.2	Yes
**Immediate postnatal care for mother:**				
Palpates uterus 15 min after delivery of placenta	557	88.3	70.2	Yes
Provider did at least one post–delivery health check	649	96.0	94.9	No
In first post–delivery exam, provider checks for bleeding	627	62.0	90.6	Yes
In first post–delivery exam, provider examines perineum	554	56.1	87.4	Yes
In first post–delivery exam, provider takes temperature	638	60.0	40.3	Yes
In first post–delivery exam, provider takes blood pressure	642	74.6	48.3	Yes
In first post–delivery exam, provider checks for involution	615	64.2	78.2	Yes
Woman asked for pain relief medication while at facility	638	32.1	10.5	Yes
Woman received pain relief medication	640	59.4	17.5	Yes
**Maternal and infant outcomes:**				
Cesarean section (C/S) performed	651	13.5	13.4	Yes
Decision for C/S taken before labor started	76	9.2	0.0	No
(Of women who had a C/S) C/S performed after labor started	76	90.8	100.0	No
(Of women who had a C/S) Provider decided C/S would be done	80	82.5	100.0	No
(Of women who had a C/S) Reason for C/S–prolonged/obstructed labor	76	32.9	67.1	Yes
Complications–any:	654	44.8	11.0	Yes
–Eclampsia	654	10.9	0.3	No
–Hemorrhage	654	11.2	4.6	Yes
–Prolonged labor (>12 h)	654	23.7	3.7	Yes
–None	654	51.5	89.0	Yes
(Of women who had complications) Blood products given	72	15.3	18.1	No
Stillborn delivery	651	0.9	1.4	No

*Table presents descriptive results. The sample size per indicator varied by women’s responses to the question, and uses matched data, excluding ‘Don’t Know’ and missing responses.

^†^Skilled provider is doctor (obstetrician/gynecologist, ob–gyn), nurse/midwife or medical resident.

^‡^Asked of mothers whose babies were breathing at birth.

^§^Newborn was placed against mother’s chest after delivery.

^#^Indicator constructed from two skin–to–skin items: (1) newborn placed against mother’s chest after delivery and (2) newborn was naked on skin (not wrapped in a towel).

**Table 3:** Indicators with greater than 5% “Don’t know” responses

Respondent question	N	“Don’t know” (%)
Did the health provider(s) wash his/her hands with soap and water or use antiseptic before examining you?	662	29.5
Was your baby dried off with a towel or cloth immediately after his/her birth, within a few minutes of delivery?	660	8.3
(Of women who reported newborn was not placed against her chest immediately after delivery) Was your baby wrapped in a towel or cloth immediately after birth?	170	20.6
In your first physical examination after delivery, did a health provider do a perineal exam?	662	9.8

Of the 41 indicators with at least five cases in each cell of the two–by–two table, four had both high individual reporting accuracy and low population–level bias (**Table 4**). These were: the main provider during delivery was a nurse/midwife, a support companion was present during the birth, cesarean operation, and low birthweight infant (<2500 g). Receiving an episiotomy was close to meeting both criteria (AUC = 0.87, 95% CI = 0.83–0.89, IF = 1.26).

**Table 4:** Validation results for indicators*

Indicator	Total number	Observer prevalence (%)	Sensitivity (95% CI)	Specificity (95% CI)	Self–report survey estimate (%), based on sensitivity and specificity	AUC (95% CI)	IF	High accuracy (AUC>0.70) & low bias (IF 0.75–1.25)
**Initial client assessment:**								
Woman referred to facility because of a problem	655	7.9	25.0 (14.0–38.9)	93.2 (90.9–95.1)	8.2	0.59 (0.55–0.62)	1.04	IF
Provider washes hands with soap and water or uses antiseptic before initial examination	467	26.6	83.9 (76.2–89.9)	32.9 (28.0–38.2)	71.5	0.58 (0.54–0.63)	2.69	
Takes blood pressure	654	87.0	87.7 (84.9–90.2)	23.3 (11.8–38.6)	86.3	0.55 (0.52–0.59)	0.99	IF
**Provider respectful care:**								
Woman allowed to drink liquids or eat	624	42.0	72.5 (66.7–77.8)	37.3 (32.3–42.5)	66.8	0.55 (0.51–0.59)	1.59	
Encourages/assists woman to ambulate during labor	644	77.5	90.2 (87.2–92.6)	24.1 (17.4–31.9)	87.0	0.57 (0.53–0.61)	1.12	IF
Encourages/assists woman to assume different positions in labor	649	58.2	19.1 (15.2–23.4)	92.3 (88.4–95.1)	14.3	0.56 (0.52–0.59)	0.25	
Woman allowed to have a support person during labor and delivery	648	9.1	23.7 (13.6–36.6)	92.7 (90.3–94.7)	8.8	0.58 (0.54–0.62)	0.97	IF
Support companion present during birth	644	4.8	48.4 (30.2–66.9)	98.5 (97.2–99.3)	3.7	0.73 (0.70–0.77)	0.77	Both
**First stage of labor:**								
Induces labor with uterotonic	630	4.6	69.0 (49.2–84.7)	92.0 (89.6–94.1)	10.8	0.80 (0.77–0.84)	2.35	AUC
Augments labor with uterotonic	625	22.4	72.9 (64.7–80.0)	70.5 (66.2–74.5)	39.2	0.72 (0.68–0.75)	1.75	AUC
Uterotonic received (labor induction or augmentation)	619	27.1	78.0 (70.9–84.0)	69.0 (64.5–73.2)	43.8	0.73 (0.70–0.77)	1.61	AUC
Membranes ruptured (labor induction or augmentation)	650	42.3	4.0 (2.0–7.0)	97.6 (95.5–98.9)	3.1	0.51 (0.47–0.55)	0.07	
**Skilled birth attendance:**								
Skilled main provider† labor	649	92.6	90.5 (87.9–92.7)	16.7 (7.5–30.2)	90.0	0.54 (0.50–0.58)	0.97	IF
–Main provider labor nurse/midwife	649	92.1	81.1 (77.7–84.2)	27.5 (15.9–41.7)	80.4	0.54 (0.50–0.58)	0.87	IF
Skilled main provider† delivery	644	92.9	95.0 (92.9–96.6)	15.2 (6.3–28.9)	94.3	0.55 (0.51–0.59)	1.02	IF
–Main provider delivery doctor (ob–gyn)/medical resident	644	11.8	82.9 (72.5–90.6)	89.3 (86.4–91.7)	19.3	0.86 (0.83–0.89)	1.63	AUC
–Main provider delivery nurse/midwife	644	81.1	86.2 (82.9–89.0)	73.0 (64.2–80.6)	75.0	0.80 (0.76–0.83)	0.93	Both
Main provider delivery student nurse	644	4.8	16.1 (5.5–33.7)	98.5 (97.2–99.3)	2.2	0.57 (0.53–0.61)	0.45	
**Second and third stage labor:**								
Episiotomy performed	545	18.2	82.8 (73.9–89.7)	90.4 (87.2–92.9)	22.9	0.87 (0.83–0.89)	1.26	AUC
Uterotonic received following delivery of placenta	552	2.4	53.9 (25.1–80.8)	40.8 (36.6–45.1)	59.1	0.47 (0.43–0.52)	25.1	
**Immediate newborn postnatal care:**								
Baby given to mother immediately after birth	611	57.6	66.5 (61.3–71.4)	49.0 (42.8–55.3)	59.9	0.58 (0.54–0.62)	1.04	IF
Baby placed immediately skin to skin on mother	602	16.3	78.6 (69.1–86.2)	21.0 (17.6–24.9)	78.9	0.50 (0.46–0.54)	4.85	
Baby placed immediately skin to skin on mother (2 item)†	596	16.2	26.8 (18.3–36.8)	70.3 (66.1–74.3)	29.2	0.49 (0.44–0.53)	1.80	
Breastfeeding within first hour of birth	551	53.0	88.4 (84.1–91.8)	37.1 (31.2–43.3)	76.4	0.63 (0.59–0.67)	1.44	
3 elements of essential newborn care (immediately dried, on mother’s skin, breastfed within first hour)	506	9.3	70.2 (55.1–82.7)	28.3 (24.2–32.7)	71.5	0.49 (0.45–0.54)	7.70	
3 elements of essential newborn care (immediately dried, 2 item on mother’s skin‡, breastfed within first hour)	501	9.2	19.6 (9.4–33.9)	74.3 (70.0–78.2)	25.2	0.47 (0.42–0.51)	2.7	
Low birthweight newborn (<2500g)	579	7.8	71.1 (55.7–83.6)	98.7 (97.3–99.5)	6.7	0.85 (0.82–0.88)	0.87	Both
**Immediate postnatal care for mother:**								
Palpates uterus 15 min after delivery of placenta	557	70.2	88.8 (85.2–91.7)	12.7 (8.0–18.7)	88.3	0.51 (0.46–0.55)	1.26	
First post–delivery exam, provider ask/checks for bleeding	627	90.6	59.9 (55.7–63.9)	17.0 (8.4–29.0)	62.0	0.38 (0.35–0.42)	0.68	
First post–delivery exam, provider examines perineum	554	87.4	57.9 (53.3–62.3)	55.7 (43.3–67.6)	56.1	0.57 (0.53–0.61)	0.64	
First post–delivery exam, provider takes temperature	638	40.3	88.1 (69.3–80.3)	50.1 (45.0–55.3)	60.0	0.63 (0.59–0.66)	1.49	
First post–delivery exam, provider takes blood pressure	642	48.3	88.1 (85.1–92.5)	38.0 (32.3–43.5)	74.6	0.63 (0.59–0.67)	1.55	
First post–delivery exam, provider checks for involution	615	78.2	64.0 (59.6–68.3)	35.1 (27.0–43.8)	64.2	0.50 (0.46–0.54)	0.82	IF
Woman asked for pain relief medication during stay	638	10.5	35.8 (24.5–48.5)	68.3 (64.3–72.1)	32.1	0.52 (0.48–0.56)	3.06	
Woman received pain relief medication	640	17.5	85.7 (77.8–91.6)	46.2 (41.9–50.6)	59.4	0.66 (0.62–0.70)	3.39	
**Maternal outcomes:**								
Cesarean section (C/S) performed	651	1.4	93.1 (85.6–97.4)	98.8 (97.5–99.5)	13.5	0.96 (0.94–0.97)	1.01	Both
Reason for C/S– prolonged/obstructed labor	76	67.1	39.2 (25.8–53.9)	80.0 (59.3–93.2)	32.9	0.60 (0.47–0.70)	0.49	
Complications (any):	654	11.0	62.5 (50.3–73.6)	57.4 (53.3–61.4)	44.8	0.60 (0.56–0.64)	4.07	
–Hemorrhage	654	4.6	33.3 (17.3–52.8)	89.9 (87.3–92.2)	11.2	0.62 (0.58–0.65)	2.43	
–Prolonged labor	654	3.7	50.0 (29.1–70.9)	77.3 (73.8–80.5)	23.7	0.64 (0.60–0.67)	6.46	
–None	654	89.0	53.8 (49.6–57.9)	66.7 (54.6–77.3)	51.5	0.60 (0.56–0.64)	0.58	

CI – confidence interval; IF – inflation factor; AUC – receiver operating curve

Recommended indicators met both AUC and IF validation criteria.

*Validation analysis based on matched data, excluding ‘Don’t Know’ responses. Sensitivity and specificity analysis was not performed for indicators that had fewer than 5 counts per cell.

†Skilled provider includes doctor (ob–gyn), medical resident or nurse/midwife.

‡Indicator constructed from two skin–to–skin items: (1) newborn placed against mother’s chest after delivery and (2) newborn was lying naked against the mother’s chest.

A total of 8 indicators had high individual reporting accuracy (AUC>0.70), 7 had moderate accuracy (AUC>0.60), and 26 had low accuracy (AUC<0.60). Indicators with high AUC results reflected events leading up to (eg, induction or augmentation of labor, episiotomy) and during the birth itself (eg, cesarean section, main provider during delivery was a doctor or medical resident, main provider during delivery was a nurse/midwife, support person present during birth, low birthweight infant). Indicators with low value AUC results tended to be related to events immediately following the birth (eg, uterotonic received following delivery of the placenta) and postnatal health checks for the mother and newborn. For population–level bias, a total of 13 indicators had low bias (0.75<IF<1.25), 7 had moderate bias (0.5<IF<1.5), and 21 had large bias (IF<0.5 or IF>1.5). Indicators with large bias varied with respect to phase of labor and delivery, but those with the largest bias tended to have a low observed prevalence and be those that may require medical knowledge to report accurately (eg, experience of complications).

To assess women’s ability to recall the type of provider who attended them, respondents were asked, “*Who was the main provider assisting you during delivery?”* There were sufficient cell counts to assess two provider categories with robust analysis: nurse/midwife and student nurse. The nurse/midwife indicator met both the high AUC and low IF criteria while the student nurse indicator had low individual accuracy (AUC = 0.57) and large bias (IF = 0.45). An indicator constructed in analysis that combines responses of “doctor”, “medical resident” and “nurse/midwife” as “skilled attendants” had low individual accuracy (AUC = 0.55), primarily due to low specificity, and low population–level bias (IF = 1.02) [28]. Cross–tabulation results that compare women’s reports to observers’ reports on their main provider during delivery suggest a tendency for medical residents and nurse/midwives to be misclassified by women as doctors (**Table 5**).

**Table 5:** Cross–tabulation of main provider during delivery based on observer reports and women’s responses

Self–report (number)	Observer report (number)							
**Doctor (obstetrician/gynecologist)**	**Medical resident**	**Medical intern**	**Nurse or midwife**	**Clinical officer**	**Student nurse**	**Other**	**Total**
Doctor (obstetrician/gynecologist)	16	46	6	46	2	7	0	123
Medical resident	0	1	0	0	0	0	0	1
Medical intern	0	0	0	1	0	1	0	2
Nurse/midwife	2	7	1	450	3	17	3	483
Clinical officer	1	0	0	12	0	1	0	14
Student nurse	0	1	0	8	0	5	0	14
Support person	0	0	0	1	0	0	0	1
No one	0	0	0	2	0	0	0	2
Other	0	2	0	2	0	0	0	4
Don’t know	1	3	0	0	0	0	0	4
**Total**	20	60	7	522	5	31	3	648

**Figure 2:**
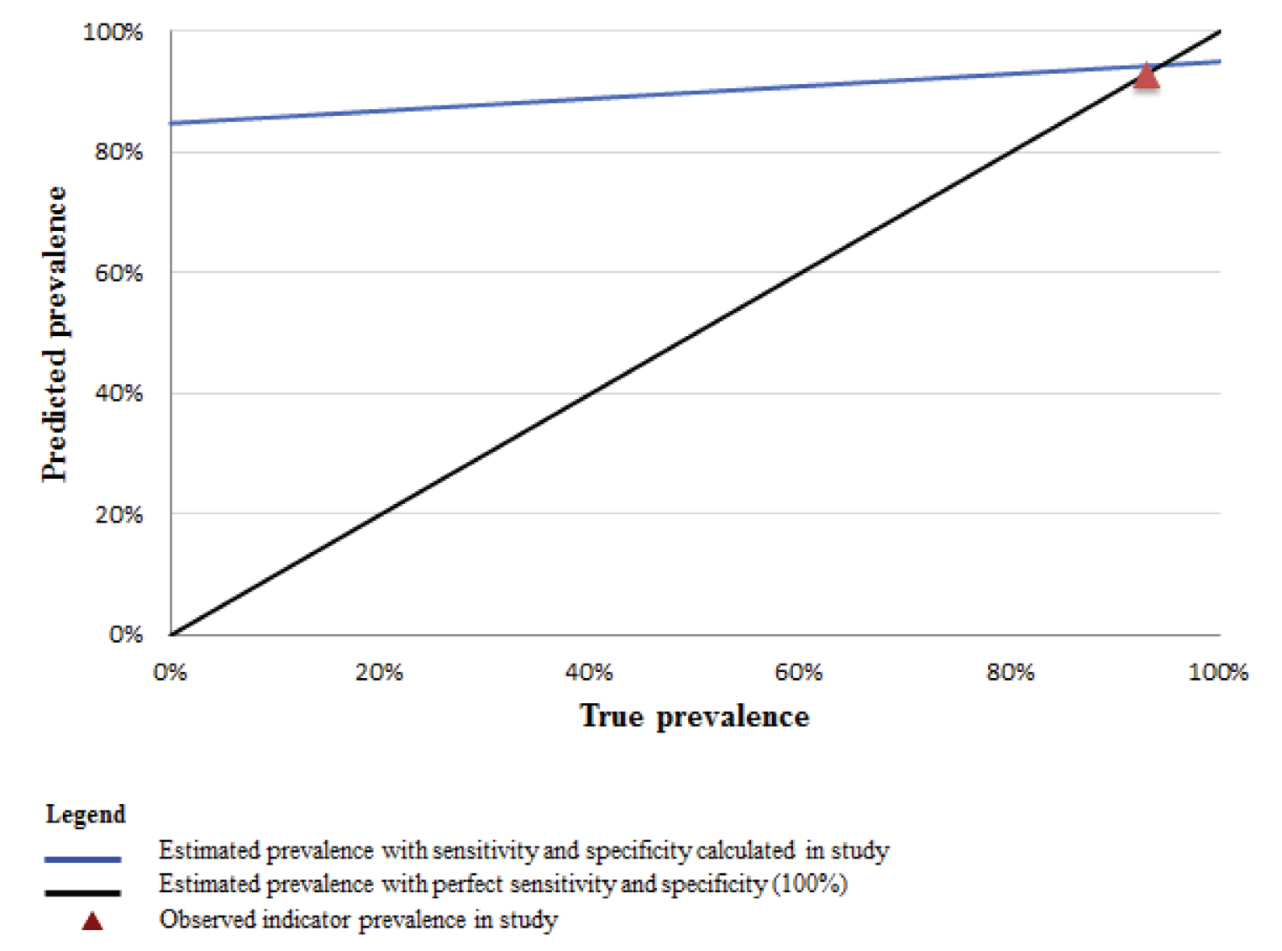
Predicted prevalence of skilled birth attendance based on sensitivity and specificity of women’s reports by true prevalence.

**Figure 3:**
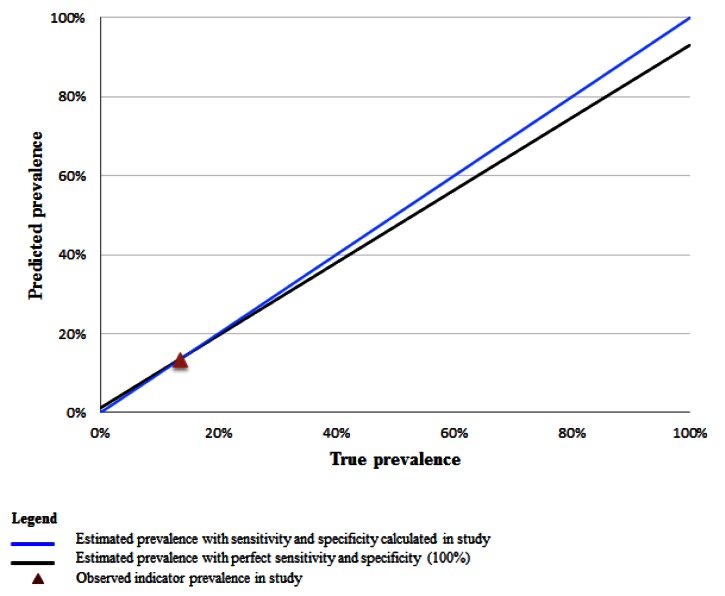
Predicted prevalence of caesarean operation based on sensitivity and specificity of women’s reports by true prevalence.

To illustrate the implications of indicator properties established in this study for other contexts, we plot the values of the predicted survey prevalence (Pr) of select indicators across all possible levels of intervention coverage (from 0 to 100%). **Figures 2** and **3** compare the predicted prevalence using the sensitivity and specificity calculated in this study (blue line), to perfect reporting accuracy assuming 100% sensitivity and specificity (black line) across all levels of coverage. Using the example of a high sensitivity and low specificity indicator such as “skilled birth attendance”, these data demonstrate that in a high coverage setting the estimated survey–based prevalence from women’s self–report more closely approximates the true prevalence while in low coverage settings, the estimated survey–based prevalence would greatly overestimate the true rate (**Figure 2**). For example, in a setting where the true prevalence of skilled attendance is 40%, rather than the 93% observed in this study (the red triangle), the estimated survey–based prevalence would exceed the true prevalence by 50 percentage points. In contrast, an indicator with both high sensitivity and high specificity, such as “cesarean operation”, would generate a survey–based estimate that closely approximates the true prevalence across all coverage levels (**Figure 3**).

## DISCUSSION

This study provides validity results for 41 indicators of the quality of maternal and immediate newborn health care that are either currently in use or have the potential to be included in household surveys. Across phases of labor and delivery, we found indicators related to concrete, observable aspects of care or which reflected pain or concern were reported with higher accuracy. These results are consistent with previous studies which have found particularly memorable events, such as having a cesarean operation [11,22,29] and having a support person present [22], have high overall validity.

That a small number of the initial list of 82 indicators met both validation criteria is partly due to the fact that many preventative care interventions almost always occurred, and there was insufficient variation (ie, not enough cases in each cell) for robust analysis. For many preventative care indicators we found that most women accurately reported receiving the care (ie, high indicator sensitivity). For example, an indicator that is a proxy for receiving a uterotonic for the prevention of postpartum hemorrhage (ie, if an injection, IV medication or tablets were received in the first few minutes following birth), was accurately reported by nearly all women. Although the near universal implementation of this practice limited robust analysis, these results suggest some aspects of routine care can be accurately reported. However, given that there were few instances in which standard preventative interventions were not received, unless there was almost perfect negative classification by women, these indicators also tended to have low specificity. An alternate interpretation is that the observed pattern of high sensitivity and low specificity for many preventative practices may reflect “facility reporting bias” among women based on the expectation of receiving appropriate care. This finding has also been described in a study of women’s reporting of maternal and child health care in China [11].

The potential for facility reporting bias may also be relevant for indicators on skilled birth attendance. Indicators measuring the assistance of a skilled provider had high sensitivity and low specificity for both labor and delivery. Women tended to underreport the presence of less skilled providers, such as student nurses, and over–report the presence of a doctor or obstetrician/gynecologist. The positive bias may also be due to differences in how women conceptualized who their “main” provider was. It is possible that women understood their “main” provider to be the attendant with the highest rank and who may have been deemed ‘in–charge’ of her care, while observers identified the primary provider as the attendant who administered the majority of the care to the woman.

Study findings also suggest the validity of some indicators may be dependent on context and question wording. Indicators that performed worse on the validity tests tend to be related to the timing or sequence of events, such as whether the newborn was placed skin–to–skin on the mother’s chest immediately after delivery. A two–item question sequence that clarified the precise meaning of “skin–to–skin” greatly reduced women’s overestimation of the practice compared to a one–item indicator (**Table 4**). These results are consistent with findings that women had difficulty reporting whether their newborn was placed skin–to–skin in a qualitative study of delivery and newborn care among women in Bangladesh and Malawi [30], but contrast with findings from a recent validation study in Mozambique [22]. The mixed results may be attributable to a longer recall period in the Mozambique study.

An influential aspect of the birth context was the type of delivery. Women who had a cesarean operation were less likely to be able to report on immediate newborn care than women with normal deliveries. This is reflected in high levels of “Don’t Know” responses. This finding suggests that it may be worth excluding women with cesarean sections from questions about care immediately after birth in routine household surveys.

A number of indicators performed well on the IF test only; individual–level misclassification does not inherently signify that measurement at the aggregate level will be inaccurate [25]. In studies where the goal is to estimate the approximate population–based coverage of an indicator, false positives and false negatives may balance out to produce a close approximation of population level coverage (ie, indicators that meet the IF criteria alone). Knowing if an indicator’s IF is large can inform when corrective methods may be needed to limit false positive reporting (eg, use of a two–item indicator).

Knowledge of whether an indicator is likely to be overestimated can also have significant programmatic implications. For example, where skilled birth attendance is over–reported, progress in scaling up the presence of higher cadre providers may not be as great as expected. It is important to recognize that the presence of a skilled provider is one aspect of receiving quality care, one which relies on the assumption that providers have received the necessary training to administer essential interventions and have acquired the competencies to address complications during childbirth. Additionally, even “skilled” providers may not be able to deliver adequate care if they do not have access to necessary equipment and supplies. Information on skilled attendance as reported by women should be corroborated with indicators on the content of care. When possible, we recommend that users also triangulate self–reported data on quality of care with other data sources such as information on stock–outs of essential medicines [4].

While a strength of this study is the use of direct observation as the reference standard, there are some limitations. Validation results are based on women seeking delivery in a large public hospital, and may not be generalizable to women who deliver in other types of facilities or at home. The lack of variation in hospital practices also limited the ability to analyze all of the indicators, which may have otherwise proven to be valid if we had collected data in a range of health institutions. Finally, our results inform a ‘best case’ scenario in terms of recall accuracy because women were interviewed shortly following delivery. To inform how recall changes over time, as well as to investigate women’s understanding of concepts such as who their main provider was, a follow–up study is under way to re–interview women one year after delivery.

## CONCLUSION

The measurement of the quality of maternal and newborn health care received in LMIC settings often relies on data from surveys of women. Little research has examined the validity of these indicators. The primary indicator of interest in this study–delivery by a skilled birth attendant–met validation criteria for reporting at the population level only and the results indicate that reporting accuracy may be particularly problematic where skilled birth attendance coverage is low. Indicator properties established here provide insight into contexts where indicator use is appropriate, and where modifications to data collection procedures or question construction may be warranted.
